# Serum Protein Electrophoretic in Children

**DOI:** 10.1155/2023/7985231

**Published:** 2023-03-02

**Authors:** Safaa Hadrach, Imane Benazzouz

**Affiliations:** ^1^Laboratory of Chemistry-Biochemistry, Environment, Nutrition, and Health, Faculty of Medicine and Pharmacy, Hassan II University, Casablanca 5696, Morocco; ^2^Biochemistry Laboratory, CHU Ibn Rochd of Casablanca, Morocco

## Abstract

Serum protein electrophoresis is a simple, reliable, and specific method used for separation of serum proteins. This study consisted to detect, at pediatric cases, pathological profiles of serum proteins by capillary electrophoresis and interpret any anomalies. The study was performed on 81 sera collected from pediatric subjects admitted at the Abderrahim Harouchi Children's Hospital in Casablanca. Study results revealed 72 specific pathological electrophoretic patterns for acute and chronic inflammatory response (35 children), hypogammaglobulinemia (3), polyclonal hypergammaglobulinemia (23), hypoalbuminemia (5), agammaglobulinemia (1), and other medical conditions (2). No cases of alpha-1-antitrypsin deficiency and nephrotic syndrome by electrophoresis were highlighted. Serum protein electrophoresis in children is recommended as a diagnostic technique for increasing the accuracy of the diagnosis in acute, subacute, and chronic inflammatory diseases, liver disease, and cases of immunodeficiency.

## 1. Introduction

Serum proteins represented mainly albumin, globulins, and fibrinogen. Minor fraction consists of lipoproteins, antibodies, enzymes, and hormones. In humans, the albumin is the most abundant plasma protein; it represents 55-60% of serum protein [1]. The distribution of these proteins on fractions in healthy people is relatively uniform. However, in certain conditions, there are variations in the amount of individual protein components of the serum (dysproteinemia), rarely meet higher amounts of protein, or develop the abnormal protein in response to various conditions. The decrease in serum albumin level (hypoalbuminemia) occurs in various pathological conditions and has different meanings (malabsorption, malnutrition, neoplasia, and nephrotic syndrome) [2]. The low level of serum albumin is an important prognostic indicator that is associated with increased mortality and morbidity, and it is increased in case of dehydration [3].

Deficiency of alpha-1 globulin is associated with lung disease and liver neonatal hepatitis [4, 5]; lowering the alpha-2 globulin level can be found in hepatocellular damage, haemolytic and megaloblastic anemia, acute severe pancreatitis, and disseminated intravascular coagulation syndrome. Serum level of beta-globulins in autoimmune disease decreases in cancer, acute and chronic infections, nephrosis, and hepatic diseases. The percentage of gammaglobulins decreases in hypogammaglobulinemia, agammaglobulinemia, and nephrotic syndromes [6, 7]. Gammaglobulin levels are increased in acute, chronic infections and rheumatoid arthritis, multiple myeloma, cirrhosis, and systemic lupus erythematosus [8].

Capillary electrophoresis is a reliable and simple method of separating protein fractions based on their physical properties: molecular weight, shape of the protein, and charge [9]. Several studies have shown the importance of electrophoretic fractionation of serum proteins in the diagnosis of various diseases where an abnormal protein level is found in the serum [10].

In Morocco, serum protein electrophoresis is poorly prescribed by doctors. The objective of this study consisted electrophoretic screening of the levels of serum protein fractions in the children hospitalized for various diseases in the Hospital for Children at the Abderrahim Harouchi Casablanca and determining the significance of abnormal protein levels in serum.

## 2. Experimental Part

The study was represented by 81 pediatric cases hospitalized in the Hospital for Children at the Abderrahim Harouchi Casablanca, during the period from 01.01.2018 to 01.01.2019 for various diseases, aged from 0 to 18 years. All patients included in our study or their legal representatives signed an informed consent form. The data was collected from an exploitation sheet, containing anthropometric information (age and sex), medical history, possible treatment, protein fraction values in % and in g/L, and the type of serum protein profile revealed on the EPS. The clinical information and all the necessary information concerning the pediatric cases were collected from the medical files of the patients at the level of the services concerned. 81 sera were analysed by capillary electrophoresis (Capillarys®, Sébia). The technique of capillary electrophoresis (Capillarys® (Sébia)) is based on the principle in free solution offering rapid separation or even complete automation of the analysis. The CAPILLARYS system contains 8 capillaries in parallel, which allows 8 simultaneous analyses. The injection into the capillaries of the sample is made at the anode by suction. On this system, the separation is carried out, while applying a potential difference of thousands of volts from each terminal of the capillary. Direct protein detection is performed from 200 nm to the cathode side. After washing the capillaries with a washing solution and with the buffer at basic pH, six protein fractions are obtained in this order: *γ*-globulins, *β*2 and *β*1-globulins, *α*2 and *α*1-globulins, and albumin. The qualitative reconstruction of the proteinogram is done by the software. Electrophoretic results and total protein were analyzed in correlation with the age of the patients. Dosage of serum total protein sera collected was carried out by the biuret colorimetric test on the Architecte ci-8200 automaton.

Data were analyzed using XLSTAT version 2022. Statistical procedures used to analyze the data included chi-square test, test, ANOVA, and Pearson correlation. Quantitative variables are presented as mean ± SD and qualitative variables as percentage. Differences between groups were taken into account as significant when *p* < 0.05.

## 3. Results and Discussion

**Figure 1:**
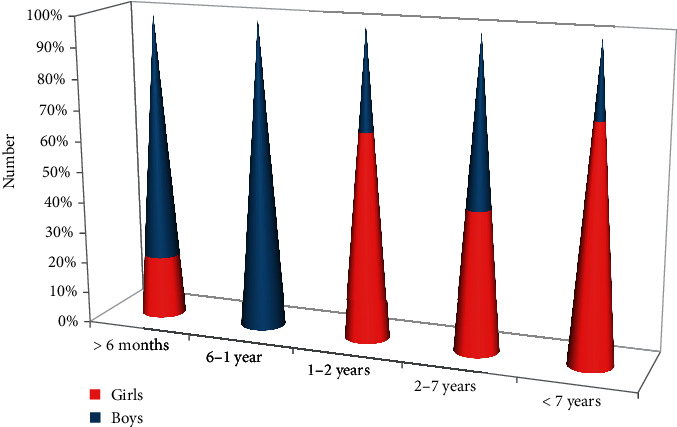
Lot distribution by age and sex.

This study included 81 pediatric patients. The average age of the pediatric patients is 4.75 ± 3.87 years. 43 cases (46.91%) were boys (4.26 ± 3.69 years old) and 38 cases (46.91%) were girls (5.36 ± 3.93 years old). The difference between sex is not being statistically significant. Pediatric patients were divided into 5 age groups: < six months, between 6 months and 1 year, between 1 year and 2 years), between 2 years and 7 years, and <7 years. The distribution of age groups indicated in Figure 1 shows a higher prevalence of children aged 2-7 years, for girls.

Accordingly, six fractions are visualized by electrophoresis: serum albumin, alpha-1-globulin, alpha-2-globulin, beta-1-globulin, beta-2-globulin, and gamma globulin. Reference values for separate protein fractions on electrophoregramme dependent on age are shown in Table 1. Interpretation of electrophoretic serum protein patterns was done according to the guide of the National College of Biochemistry of Hospitals (NCBH) which offered ready-to-use interpretative comments, concerning serum protein electrophoresis 2006 [11]. Total serum proteins were determined by the biuret colorimetric test on the Architecte ci-8200 automaton.

The results concerning at paraclinical and clinical parameters, presented in Table 2, indicate that the wide variation limits for each parameter were analyzed. A number of 72 patients have presented abnormality electrophoretic; serum levels of total protein, globulins, and albumin were not framed in reference ranges recommended for children (Table 1). Decrease in total protein corresponds to a reduction in the levels of albumin, less being influenced by the decrease or increase in globulins. Decrease in serum albumin levels was recorded in 66 cases, compared to gamma globulin levels which were high in 23 cases and lower in 4 cases. Comparative analysis between gamma globulin and the following age groups, under 6 months, between 6 months and 1 year, 1 year and 2 years, 2 years and 7 years, and over 7 years, showed a statistically significant association with a *p* value of 0.0001 < *p* − alpha (0.05). Age was significantly correlated with total protein (*r* = −0.833; *p* < 0.002) and beta-globulin (*r* = 0.244; *p* < 0.027). The children had different symptoms. The distribution of serum protein fractions according to the electrophoretic pattern is shown in Table 3. Most patients have been showing subacute inflammatory response (11.1%), followed by hypogammaglobulinemia (3.7%), acute inflammatory response (32.1%), and polyclonal hypergammaglobulinemia (28.4%), and for the rest of diseases were registered percentages under (2%).

**Figure 2:**
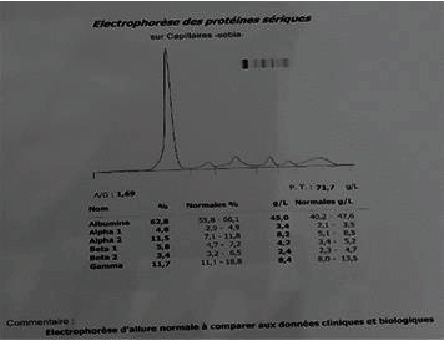
Normal electrophoretic.

**Figure 3:**
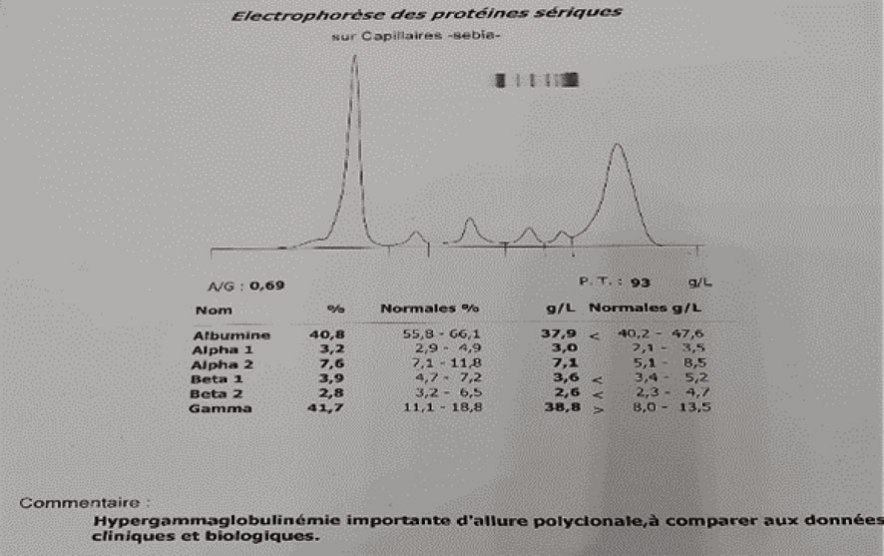
Polyclonal hypergammaglobulinemia electrophoretic.

**Figure 4:**
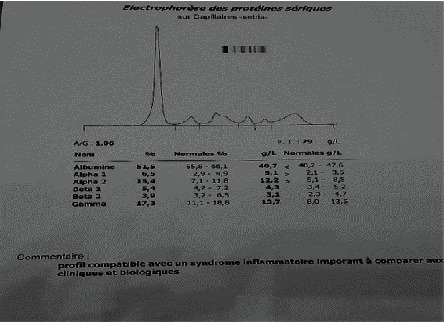
Inflammatory response electrophoretic.

In Figures 2–4, examples of electrophoretic patterns in healthy patients, with chronic and acute inflammatory response, are shown. Normally, healthy subjects present a uniform aspect of the electrophoretic profile, although the components of the serum are normal; therefore, slight variations can be perceived due to certain ethnic, genetic consequences, the diet followed, and the daily behavior of the individual. Appearance of the electrophoretic profile is altered in certain diseases and nutritional disorders. The change is generally manifested by an increase or decrease in the protein fraction. The decrease in albumin level is the main consequence of the reduction in the rate of synthesis, inflammation being known to cause the deletion of the albumin transcription gene [12]. Additionally, protein malnutrition can affect a patient's protein synthesis. The loss of plasma proteins contributes to the decrease in serum protein levels, passing through hemodialysis membranes [13].

The moderate inflammatory protein profile recorded a level of 4.48 ± 0.90 g/L, at the level of the alpha-1 globulin fraction, and a level of 9.88 ± 1.53 g/L, at the level of fraction of alpha-2 globulin. These values are slightly increased compared to the important inflammatory protein profile, where alpha-1 globulin recorded a rate of 5.16 ± 1.30 g/L at alpha-2 globulin at a rate of 12.42 ± 1.65 g/L. This explains that the proteins of inflammation increase proportionally according to the importance of the inflammation and gamma globulins recorded a rate of 13.77 ± 6.28 g/L. During an inflammatory reaction, certain serum proteins undergo a slight imbalance. It is either loss or synthesis of proteins. Indeed, during an acute inflammation, the liver synthesizes inflammation proteins called APR (acute phase reactants) [14]. Generally, alpha-1 antichymotrypsin, alpha-1 antitrypsin, and alpha-1 acid glycoprotein (orosomucoid) migrate in the alpha-1 globulin fraction. Ceruloplasmin and haptoglobin migrate to the alpha-2 globulin fraction, and C-reactive protein (CRP) migrates to the gamma globulin fraction [15]. These fractions increase proportionally during the inflammatory syndrome [16]. A hypoalbuminemia of 33.8 g/L was noted in a ten-month-old girl representing a recurrent lung infection, a lower value according to the references for her age group. In fact, several studies show that hypoalbuminemia leads to pleural or pericardial effusions [17]. Also, hypoalbuminemia is an ignored and changeable risk factor that contributes to the onset of cardiovascular and pulmonary diseases. The serum albumin assay is very important and can be a tool for any cardiovascular and pulmonary disease [18].

In children with hypergammaglobulinemia, albumin is 31.02 ± 7.37 g/L; the values of *α*-1 globulins, *α*-2 globulins, and *β*-globulins are normal, and an increase in the level of the *γ*-globulin fraction is 23.10 ± 7.00 g/L, whereas a study in Romania in pediatric cases revealed a lower increase in *γ*-globulins of the order of 18.4 ± 4.9 g/L [19]. The highest concentration of *γ*-globulins was recorded in girls with a rate of 23.22 g/L, against 22.98 g/L in boys. The protein profile of hypergammaglobulinemia in our study was statistically significant with gender (*p* = 0.007). This agrees with the study that was performed by Gurau et al. in 2016, which shows that the level of *γ*-globulins is higher in girls by 2.49 g/dL, compared to 2.19 g/dL in boys [19]. Lo et al. suggest that girls with high levels of IgG are three times more at risk for autoimmune diseases than boys [20]. Polyclonal hypergammaglobulinemia is due to the activation of a large number of plasma cell clones which release immunoglobulins. It is observed on a serum protein profile during a homogeneous and symmetrical separation of gammaglobulins, giving the appearance of a Gaussian curve. The increase in the fraction of gamma globulin is justified by the mutation of NK cells (natural killer) and cytotoxic T lymphocytes (CTL) preventing the lysis of macrophages and continuing to produce a large quantity of gamma [21].

The hypergammaglobulinemia serum protein profile of 28 cases in our series was associated with hepatitis A in 30.34%, autoimmune hepatitis in 17.39%, and autoimmune thrombocytopenic purpura in 8.69%. Hypergammaglobulinemia can reach an extreme value of 38.86 g/L, which is considered the highest level detected in a 10-year-old girl with prolonged hepatitis.

Hypogammaglobulinemia is a type of primary immune deficiency. The concentration of gammaglobulins is manifested by a decrease in the blood, in particular IgG and IgA [22].

Hypogammaglobulinemia was recorded only in 3 children (3.7%) aged 0.24 ± 0.19 years. The average level of *γ*-globulin is 5.40 ± 0.53 g/L, a value below the minimum limit indicated in the age group of these children. So, the study made by Gurau et al. recorded a hypogammaglobulinemia of 4.1 ± 1.5 g/L [19]. Most hypogammaglobulinemic children have a history of recurrent infections, mainly during primary immunodeficiency [23].

Our study recorded three pediatric cases, the first of which is a 2-month-old child who presents with generalized jaundice from the 4th day of life, with dark urine and monocolor stool currently afebrile with a cholestatic syndrome. The second child is 48 days old, also presenting with jaundice, and the third child presents with prolonged free bilirubin jaundice. It is possible that these patients to submit transient hypogammaglobulinemia when IgA and IgG levels can remain low in the first six months after birth, either are not yet produced or the formation of antibodies is prevented because of malnutrition [24]. Hypogammaglobulinemia is associated with various pathological disorders, such as gastroenteritis, jaundice, nonspecific fever, intestinal and urinary infection, digestive function disorder, allergy, liver damage, and malnutrition [25].

Hypoalbuminemia is an abnormality characterized by a decrease in the level of albumin. It is moderate when the albumin level is between 30 g/L and 35 g/L, and it is significant when the albumin level is less than 30 g/L. Hypoalbuminemia is often attributed to malabsorption, malnutrition, hepatic dysfunction, nephropathy, or protein-losing enteropathy. Also, hypoalbuminemia is present in the case of inflammation by the acceleration of the catabolism of albumin which decreases its production, consequently the stimulation of the synthesis of reactive proteins of the acute phase, and this is possibly another cause of hypoalbuminemia in critically ill patients [26].

In our series, hypoalbuminemia was recorded in 5 children (6.2%), aged 6.00 ± 3.79 years, and then, in the study carried out by Gurau et al., the average age of the child is 10.04 ± 6.68 years in hypoalbuminemia patients [19]. A hypoalbuminemia of 30.90 ± 3.26 g/L, with a male predominance was recorded in our series. These results are almost similar with the study which examines the association of hypoalbuminemia and critically ill children and which reported a hypoalbuminemia of 26.1 g/L ± 6.7 g/L, in a predominant population of the sex male [27]. So, the study by Gurau et al. recorded a hypoalbuminemia of 28.7 ± 2.8 g/L in children who suffer from respiratory failure with a longer stay in the hospital [19].

The variation in the protein profile of hypoalbuminemia from one country to another can be explained by the dietary habits in the country as well as by the socioeconomic level of the patients, or perhaps it is related to the degree of severity of the disease. The two pathologies that have been associated with hypoalbuminemia in our series are visceral leishmaniasis and storage disease.

Only one case of agammaglobulinemia has been recorded, in a 3-year old boy. In order to confirm that a child has humoral immunodeficiency, it is necessary to eliminate certain nephrotic symptoms and protein-losing enteropathies, hematological malignancies, and also the use of certain drugs, in particular immunosuppressants [28]. In our case, the only information we could retain about the child is that he has diffuse interstitial lung disease. So, we are not sure if this agammaglobulinemia is linked to a humoral immune deficiency or simply to other physiological mechanisms (nephrotic syndrome, enteropathy, immunosuppressants, etc.). All types of humoral immunodeficiency, namely, congenital agammaglobulinemia, typical iso immunoglobulin class switching defect, common variable immunodeficiency, transient hypogammaglobulinemia in children, and selective deficiency of IgA, IgG2, and antipolysaccharide antibodies, show signs associated with respiratory infections, ENT infections, or recurrent respiratory [28]. One study showed that 80% of children who have immunoglobulin class-typical iso-switching humoral immunodeficiency develop bacterial pneumonia [29].

In general, children with agammaglobulinemia are more susceptible to severe lung diseases, which cause chronic obstructive complications in the lungs, bronchi, and recurrent pneumonia [30].

Serum protein electrophoresis, although considered a second-line diagnostic examination, allows the orientation of the different pathological serum protein profiles, namely, the inflammatory protein profile, the immunological protein profile, the protein profile nephrotic, the protein profile of hypoalbuminemia, or the protein profile cirrhotic, by the analysis of the different proteins.

Serum protein electrophoresis is a well-known laboratory technique but rarely recognized as an important and necessary diagnostic method for clinical routine. The serum is the source of thousands of proteins, but only 22, including alpha-1-antitrypsin, are high enough for their changes to affect the electrophoregram profile. Our study did not record any pediatric cases of alpha-1 antitrypsin deficiency.

However, this means that the variability of the concentration of individual proteins in the serum, including the opinion about alpha-1-antitrypsin deficiency, may be premature only on the basis of electrophoregram and requires additional specific confirmation methods.

## 4. Conclusion

Serum protein electrophoresis allows the pediatrician to identify different serum proteins, in order to guide the diagnosis and to evaluate the maintenance of the various diseases and also to ensure the follow-up and to measure the therapeutic effectiveness. Serum protein electrophoresis should be a routine laboratory test applied to all pediatric cases on admission, as it can provide valuable clues to the severity, cause, and location of pathology.

## Figures and Tables

**Table 1:** Reference ranges for total protein and protein fractions isolated through electrophoresis, by age groups.

Age (years)	Serum total protein g/L	Albumin % (g/L)	*α*-1 G % (g/L)	*α*-2 G % (g/L)	ß-1 G % (g/L)	*β*-2 G % (g/L)	*γ* G % (g/L)
<6 months	64.5	58.9-73.4 27.3-49.1	3.2-11.7 2.1-5.4	10.6-14 5.3-9.8	4.8-7.9 2.2-4.6	2.1-3.3 1.1-2.1	3.5-9.7 1.7-6.3
6-1 year	70	57.4-71.4 36-50.6	3-5 2-3.7	10.2-16.1 6.3-12.1	5.3-6.9 3.3-4.9	2.2-3.6 1.4-2.6	4.2-11 2.8-8
1-2 years	72.5	57.4-69 38.7-51.1	3.2-5.4 2.4-4	10.7-15.5 7.8-11.6	5.6-7 3.7-5.2	2.6-4.2 1.5-3.1	7.7-14.8 4.6-10.7
2-7 years	71	57.5-67.7 30.5-48.9	3.2-5.4 2-3.7	10-14.8 5.6-10.6	5.1-6.9 2.7-5.2	2.9-5.2 1.7-3.9	9.8-16.9 6-12.7
>7 years	73	57.1-67.2 30.9-49.5	3.2-4.9 1.7-3.7	8.9-13 4.8-9.7	5.1-6.9 2.7-5.2	2.9-5.2 1.7-3.9	9.8-16.9 6-12.7

**Table 2:** Paraclinical and clinical parameters of the subjects in study group.

Parameters	No	Mean	Minim	Maxim	SD^∗^
Age (years)	81	5.02	0.00	17.00	3.95
Albumin (g/L)	81	35.58	16.10	52.70	7.28
Alpha-1 globulin (g/L)	81	3.82	1.40	7.60	1.40
Alpha-2 globulin (g/L)	81	8.16	2.50	15.40	3.01
Beta-1 globulin (g/L)	81	3.84	1.20	13.70	1.50
Beta-2 globulin (g/L)	81	3.05	0.40	11.00	1.47
Gammaglobulin (g/L)	81	15.11	2.10	38.80	7.73
Report albumin/globulin (A/G)	81	1.14	0.38	2.29	0.410
Total serum protein TP (g/L)	81	69.74	45.20	102.10	11.82

SD^∗^: standard deviation.

**Table 3:** Electrophoretic pathological pattern in study group.

Electrophoretics pattern	*N* (%)^∗^	Albumin (g/L)	*α*1-globulin (g/L)	*α*2-globulin (g/L)	*β*1-globulin (g/L)	*Β*2-globulin (g/L)	y-globulin (g/L)
Acute inflammatory response	26 (32.1)	36.88 ± 5.83	4.48 ± 0.90	9.88 ± 1.53	3.95 ± 1.04	3.34 ± 1.04	13.77 ± 6.28
Subacute inflammatory response	9 (11.1)	39.60 ± 7.66	5.16 ± 1.30	12.42 ± 1.65	5.18 ± 3.10	3.30 ± 1.07	12.48 ± 4.60
Polyclonal hypergammaglobulinemia	23 (28.4)	31.02 ± 7.37	3.54 ± 1.44	6.03 ± 2.61	3.49 ± 1.10	3.05 ± 1.47	23.10 ± 7.00
Normal electrophoretics	9 (11.1)	42.58 ± 4.34	3.05 ± 0.52	6.94 ± 0.69	3.88 ± 0.75	2.74 ± 0.56	10.95 ± 2.27
Hypoalbuminemia	5 (6.2)	30.90 ± 3.26	4.69 ± 1.96	8.22 ± 2.94	3.78 ± 1.13	3.14 ± 0.95	14.22 ± 5.74
Hypogammaglobulinemia	3 (3.7)	41.03 ± 4.20	2.65 ± 0.15	7.40 ± 1.06	3.30 ± 0.53	2.06 ± 0.33	5.40 ± 0.53
Hypoprotidemia	3 (3.7)	30.93 ± 0.65	1.76 ± 0.12	3.76 ± 0.83	2.40 ± 0.63	1.43 ± 0.73	9.10 ± 2.79
Agammaglobulinemia	1 (1.2)	22.3	7.6	12.1	2.3	1.6	2.1
Other conditions	2 (2.5)	—	—	—	—	—	—

(%)^∗^: percentages reported by the total number of patients (81).

## Data Availability

All data generated or analyzed during this study are included within the article.
